# Intraoperative Diagnosis and Management of Arginine Vasopressin Disorder During Pituitary Tumor Resection via Transsphenoidal Endoscopic Navigation

**DOI:** 10.7759/cureus.82096

**Published:** 2025-04-11

**Authors:** Delayne M Coleman, Emily Kim, Krupa Patel, Richesh Guragain, Beth Teegarden

**Affiliations:** 1 Department of Anesthesiology, John Sealy School of Medicine, University of Texas Medical Branch, Galveston, USA; 2 Department of Anesthesiology, University of Texas Medical Branch, Galveston, USA

**Keywords:** antidiuretic hormone, arginine vasopressin disorders, desmopressin, pituitary adenoma, transsphenoidal surgery, urine monitoring

## Abstract

Arginine vasopressin (AVP) disorders (previously called diabetes insipidus) lead to excessive urination due to reduced antidiuretic hormone (ADH) secretion or kidney resistance to ADH. This results in decreased water reabsorption, causing dehydration and electrolyte imbalances. Diagnosing these disorders during general anesthesia is challenging, but close monitoring of electrolytes and urine output, especially during high-risk surgeries such as intracranial procedures, is crucial.

A 64-year-old woman with a history of asthma presented with severe bifrontal headaches and left-eye medial gaze palsy. Imaging showed a large sellar mass extending into the sphenoid sinus, requiring a transsphenoidal resection. An hour and 30 minutes into surgery, the patient developed acute polyuria (1 L urine), hyperosmolality (Na: 149 mmol/L), and colorless urine with low specific gravity (1.003), indicating an arginine vasopressin disorder. Desmopressin (DDAVP) was administered, improving urine specific gravity to 1.013, and a D5W infusion corrected a 2.5 L fluid deficit. Severe hypokalemia (K: 2.6 mmol/L) and hyperglycemia (glucose: 230 mg/dL) were also treated, with electrolyte and glucose levels stabilizing postoperatively. On postoperative day (POD) 2, the patient experienced polyuria up to 23 L and excessive thirst, requiring additional desmopressin on POD 3. She was discharged on POD 9. Arginine vasopressin disorders, especially vasopressin deficiency (central diabetes insipidus), commonly result from neurohypophyseal damage during cranial surgery. Prompt diagnosis and treatment with desmopressin and fluids can effectively manage fluid and electrolyte imbalances, preventing severe complications such as hypernatremia and neurological deficits. This case highlights the importance of intraoperative urine and laboratory monitoring to ensure timely recognition and management.

## Introduction

Pituitary adenomas are commonly benign tumors that originate from the adenohypophysis and often cause clinical symptoms by expanding and compressing nearby structures [1]. Currently, transsphenoidal resection is one of the most common surgical techniques for pituitary adenomas, especially when patients present with visual disturbances, significant rates of growth, or loss of endocrine function. Transsphenoidal resection is known for its minimally invasive approach, mild postoperative symptoms, and quick recovery. However, it frequently results in mechanical damage to the hypothalamic-neurohypophyseal system, leading to the development of arginine vasopressin (AVP) disorders, formerly known as diabetes insipidus [2].

The hypothalamic-neurohypophyseal system consists of the hypothalamus and the neurohypophysis (posterior pituitary), which form a complex that works together to regulate essential hormones. The neurohypophysis is responsible for the secretion of oxytocin and antidiuretic hormone (ADH), whose role is to prevent diuresis. These hormones are produced by the magnocellular neurosecretory cells of the hypothalamus before being transported to the neurohypophysis, where they are stored until later system distribution by the neurohypophyseal capillaries in response to changes in volume and osmolarity [3].

In this report, we discuss the intraoperative diagnosis and management of transient arginine vasopressin deficiency associated with severe electrolyte disturbances.

This case was presented as a poster at the Texas Society of Anesthesiologists Annual Meeting in September 2024 and the American Society of Anesthesiologists Annual Meeting in October 2024.

## Case presentation

**Figure 1:**
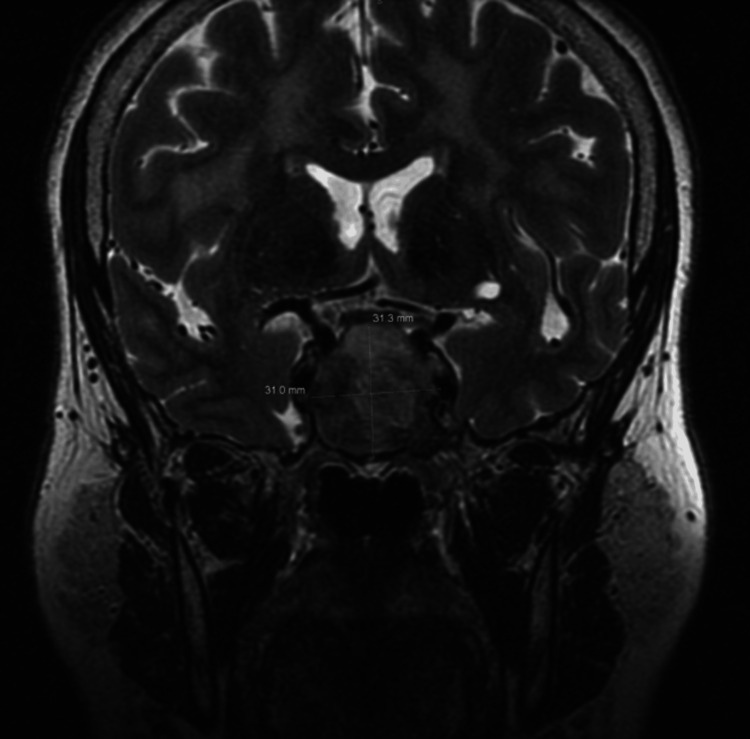
MRI findings portraying a large pituitary macroadenoma measuring approximately 4 cm anteroposteriorly, 3.1 cm side to side, and 3.1 cm cephalocaudally, with extension into the sphenoid sinus MRI: magnetic resonance imaging

A 64-year-old woman with a history of asthma presented with three days of new-onset severe bifrontal headaches and left-eye medial gaze palsy. Laboratory tests initially showed decreased prolactin, morning cortisol, and thyroid-stimulating hormone (TSH). Imaging revealed a large expansile sellar mass measuring approximately 4 cm anteroposteriorly, 3.1 cm side to side, and 3.1 cm cephalocaudally, with extension into the sphenoid sinus, suggestive of a pituitary macroadenoma with apoplexy. The decision was thus made to perform transsphenoidal sellar mass resection by otolaryngology and neurosurgery (Figure 1). General endotracheal anesthesia was induced without complication. Intraoperatively, an arterial line and a central line were placed with ultrasound guidance. Notable intraoperative medications include 100 mg IV hydrocortisone and no diuretic (Lasix or mannitol) administration.

**Figure 2:**
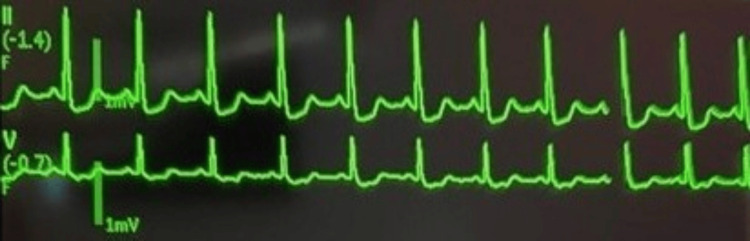
Intraoperative EKG demonstrating mild ST depression and u-wave inversions consistent with hypokalemia EKG: electrocardiogram

About 1.5 hours into the surgery, the patient developed acute and persistent polyuria with 1 L clear urine documented and hyperosmolality (Na: 149 mmol/L on arterial blood gas (ABG) analysis, normal range: 136-146 mmol/L) (Table 1). Urine studies obtained 3.5 hours from the start of the case revealed colorless urine with a low urine specific gravity of 1.003 (normal range: 1.005-1.030) and pH of 8 (normal range: 4.6-8), suggesting dilute urine and confirming the suspected diagnosis of an arginine vasopressin (AVP) disorder (Table 1). Treatment was initiated with desmopressin (DDAVP), and repeat intraoperative urine studies performed 30 minutes later demonstrated an improvement in urine specific gravity to 1.013 (Table 1). A D5W infusion was required to correct the free water deficit of 2.5 L. Electrolyte abnormalities noted during this time were severe hypokalemia of 2.6 mmol/L (normal range: 3.5-5 mmol/L), associated with mild ST depression and u-wave inversions on electrocardiogram (EKG), and hyperglycemia (glucose: 230 mg/dL, normal range: <140 mg/dL) requiring insulin therapy (Table 1, Figure 2). On subsequent ABG analysis, Na downtrended to normal, and K improved with repletion.

**Table 1:** Intraoperative laboratory findings demonstrating fluid, metabolic, and electrolyte imbalances before and after desmopressin administration

Parameters	Reference range	Patient values before desmopressin	Patient values after desmopressin
Serum Na+	136-146 mmol/L	149 mmol/L	146 mmol/L
Serum K+	3.5-5.0 mmol/L	2.8 mmol/L	2.6 mmol/L
Serum glucose	Random, non-fasting: <140 mg/dL	157 mg/dL	230 mg/dL
Urine specific gravity	1.005-1.030	1.003	1.013
Urine pH	4.6-8	8	7

Postoperative, endocrinology continued to monitor her fluid balance and initiated sliding scale insulin with Lispro to manage hyperglycemia. The patient was placed on strict intake and output monitoring with urine output checks every hour. Serum sodium and urine specific gravity checks were ordered every 12 hours. On postoperative day (POD) 2, the patient continued to experience polyuria with urine output volumes up to 23 L and polydipsia with fluid intake >20 L. Serum sodium at this time was 141 mmol/L (normal range: 136-146 mmol/L) and urine specific gravity was 1.004 (normal range: 1.005-1.030) (Table 2), requiring an additional dose of desmopressin on POD 3. Subsequently, the patient began to demonstrate stable oral intake and urine output and was discharged home on POD 9.

**Table 2:** Postoperative course laboratory findings demonstrating fluid and electrolyte imbalances DDAVP: desmopressin

Date	Fluid input (24 hours) (mL)	Urine output volume (24 hours) (mL)	Serum Na range (normal range: 136-146 mmol/L)	Urine specific gravity range (normal range: 1.005-1.030)	Desmopressin
3/21	3,802	7,460	138-145	1.003-1.003	DDAVP 1 mcg IV in NaCl 0.9% (NS) piggyback
3/23	18,531	23,260	136-139	1.002-1.006	-
3/24	22,973	21,470	136-139	1.002-1.005	-
3/25	-	-	135-141	1.004-1.019	DDAVP 0.04 mcg SQ
3/26	2,171	6,450	135-138	1.005-1.005	-
3/27	9,335	3,966	132-135	1.004-1.005	-
3/28	1,750	4,635	136-146	1.006-1.007	-

## Discussion

AVP disorders result in polyuria from either decreased secretion of antidiuretic hormone (ADH) or resistance to its effects on the kidney [4]. These symptoms can lead to electrolyte imbalances and varying levels of dehydration, which may become life-threatening in severe cases. The incidence of AVP-D, the deficiency variant, after pituitary adenoma surgery ranges from 9% to 22% [5] and is usually linked to an intraoperative injury to the hypothalamus or pituitary stalk. Researchers have examined several clinical factors, including gender, age, serum pituitary hormone levels, and intraoperative cerebrospinal fluid leakage, to identify potential predictors of AVP-D, although consensus has yet to be reached [6]. From an epidemiological perspective, AVP does not affect males or females preferentially and can arise at any age, with hereditary forms typically emerging earlier in life [7].

Diagnosing these disorders under general anesthesia remains challenging and requires a preoperative understanding of high-risk procedures and patients at risk, utilization of diagnostic tests, and management strategies. AVP is more likely to occur in neurosurgical procedures or after head trauma, especially those involving the pituitary or the hypothalamus. Patients with a pituitary mass face a higher risk, particularly if the mass is large, extends into the suprasellar space, or causes visual disturbances [8]. The presence of these risk factors should prompt planning for potential AVP onset. This preparation includes monitoring for increasing high-volume urine output and the ability to order the appropriate laboratory tests. Standard laboratory work for AVP diagnosis includes urinalysis, electrolyte monitoring, and assessments of serum and urine osmolality [4]. The differential for polyuria ranges from primary medication-induced, glucosuria, and large-volume infusions. Diagnosing AVP can be done systematically once polyuria has been identified by confirming the tonicity of the urine and, subsequently, the plasma. AVP will manifest with a low urine osmolality (<300 mOsm/kg) and a low urine specific gravity (<1.003). Conversely, the plasma osmolality will be elevated (≥300 mOsm/kg) with high serum sodium (>146 mmol/L). If AVP is diagnosed, treatment should begin immediately.

The mainstay of therapy for AVP deficiency is desmopressin (DDAVP) [4]. Desmopressin is an ADH analog and thus binds to the aquaporin-2 channels in the collecting duct in the kidney to increase water reabsorption [9]. In addition to desmopressin, it is imperative to replenish fluid losses, particularly in patients with impaired thirst or who are unable to tolerate oral fluids.

Serum sodium should be rechecked every four hours during the acute treatment phase to monitor for signs of overtreatment, such as hyponatremia, which can potentially lead to cerebral edema and herniation [10]. Current guidelines recommend that the rate of hypernatremia correction should not exceed 0.5 mmol/L per hour or 10 mmol/L per day. However, evidence suggests that in cases of severe hypernatremia, the risk of adverse neurological complications is low. Therefore, physicians should use clinical judgment to determine the optimal correction rate in these situations [11]. To avoid hyponatremia, repeat doses of desmopressin should be limited to the effective dose needed to manage polyuria, typically 0.25-1 mcg of IV or SQ desmopressin every 12-24 hours [12]. Additionally, while most AVP episodes are transient, lasting one week to three months, there is a possibility of permanence [8]. Thus, regular follow-up to monitor for symptom resolution is integral in assessing the course of the illness.

## Conclusions

Arginine vasopressin disorders, particularly the formerly referred to as central variant (vasopressin deficiency), can commonly result from damage or disruption to the neurohypophysis, especially during cranial surgery. After diagnosis, treatment with desmopressin, an antidiuretic hormone analog, and fluids can effectively correct fluid and electrolyte derangements. This case illustrates the significance of preoperative planning, urine monitoring, and intraoperative laboratory studies leading to prompt diagnosis and treatment. The patient's rapid response to desmopressin and the normalization of serum sodium and potassium levels underscore the importance of early diagnosis and treatment of AVP disorders in the perioperative setting. This case highlights the need for vigilant monitoring and prompt intervention to prevent severe complications, such as severe hypernatremia and associated neurological consequences, such as seizures, coma, or focal neurological deficits.
